# Supplementary material for: Evidence supported by Mendelian randomization: impact on inflammatory factors in knee osteoarthritis
Source: Front Med (Lausanne). 2024 May 28;11:1382836. doi: 10.3389/fmed.2024.1382836 (PMC11165061; doi:10.3389/fmed.2024.1382836)
Supplement: Supplementary file 1 [file Data_Sheet_1.ZIP › data/GCST90274816_5e6/816.leaveoneout.pdf]

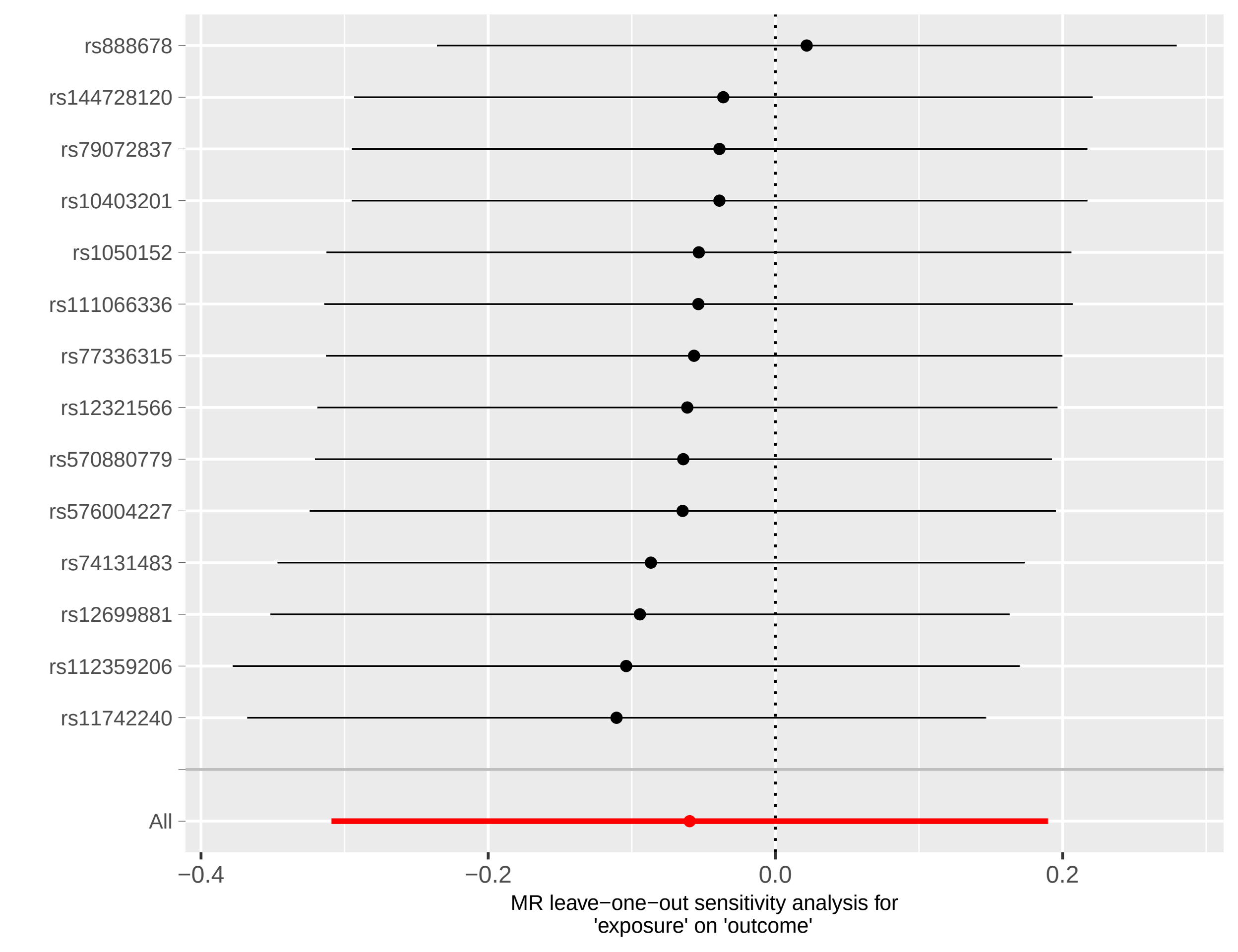
rs888678
rs144728120
rs79072837
rs10403201
rs1050152
rs111066336
rs77336315
rs12321566
rs570880779
rs576004227
rs74131483
rs12699881
rs112359206
rs11742240
All
−0.4
−0.2
0.0
0.2
MR leave−one−out sensitivity analysis for
'exposure' on 'outcome'
